# Nocturnal Foraging by Red-Legged Kittiwakes, a Surface Feeding Seabird That Relies on Deep Water Prey During Reproduction

**DOI:** 10.1371/journal.pone.0138850

**Published:** 2015-10-14

**Authors:** Nobuo Kokubun, Takashi Yamamoto, Dale M. Kikuchi, Alexander Kitaysky, Akinori Takahashi

**Affiliations:** 1 National Institute of Polar Research, 10–3 Midori-cho, Tachikawa, Tokyo, 190–8518, Japan; 2 Department of Polar Science, The Graduate University for Advanced Studies (SOKENDAI), 10–3 Midori-cho, Tachikawa, Tokyo, 190–8518, Japan; 3 Graduate School of Fisheries Sciences, Hokkaido University, 3-1-1 Minato-cho, Hakodate, Hokkaido, 041–8611, Japan; 4 Institute of Arctic Biology, Department of Biology and Wildlife, University of Alaska Fairbanks, Irving 311, Fairbanks, Alaska, 99503, United States of America; Phillip Island Nature Parks, AUSTRALIA

## Abstract

Narrow foraging specialization may increase the vulnerability of marine predators to climate change. The red-legged kittiwake (*Rissa brevirostris*) is endemic to the Bering Sea and has experienced drastic population fluctuations in recent decades, presumably due to climate-driven changes in food resources. Red-legged kittiwakes are presumed to be a nocturnal surface-foraging seabird that feed almost entirely on deep water *Myctophidae* fishes. However, there is little empirical evidence confirming their nocturnal foraging activity during the breeding season. This study investigated the foraging behavior of red-legged kittiwakes by combining GPS tracking, accelerometry, and dietary analyses at the world’s largest breeding colony of red-legged kittiwakes on St. George I. GPS tracking of 5 individuals revealed that 82.5% of non-flight behavior (including foraging and resting) occurred over the ocean basin (bottom depth >1,000 m). Acceleration data from 4 birds showed three types of behaviors during foraging trips: (1) flight, characterized by regular wing flapping, (2) resting on water, characterized by non-active behavior, and (3) foraging, when wing flapping was irregular. The proportions of both foraging and resting behaviors were higher at night (14.1 ± 7.1% and 20.8 ± 14.3%) compared to those during the day (6.5 ± 3.0% and 1.7 ± 2.7%). The mean duration of foraging (2.4 ± 2.9 min) was shorter than that of flight between prey patches (24.2 ± 53.1 min). Dietary analyses confirmed myctophids as the dominant prey (100% by occurrence and 98.4 ± 2.4% by wet-weight). Although the sample size was limited, these results suggest that breeding red-legged kittiwakes concentrated their foraging on myctophids available at the surface during nighttime in deep water regions. We propose that the diel patterns and ephemeral nature of their foraging activity reflected the availability of myctophids. Such foraging specialization may exacerbate the vulnerability of red-legged kittiwakes to climate change in the Bering Sea.

## Introduction

Narrow foraging specialization may increase the vulnerability of marine predators to climate change [1]. Foraging specialization in time and space characterizes the ecological niche of a predator [2]. Seabirds search for and feed on prey at sea by moving long distances from the breeding colony, and are thought to detect and capture prey in a heterogeneous marine environment that has predictable, large-scale oceanographic features (e.g. marine frontal systems [3,4]). Seabirds maximize their foraging efficiency under their morphological and physiological constraints by specializing on prey type [5], feeding method [6], foraging location or zone [7], or diel foraging pattern (nocturnal, crepuscular or diurnal [8,9]).

The red-legged kittiwake (*Rissa brevirostris*, hereafter RLKI) is endemic to the Bering Sea [10,11] and has been listed as a species of conservation concern due to its small reproductive range and decreasing population trends driven by changes in food resources [12]. The foraging ecology of the RLKI is not well studied, which limits our current understanding of bottom-up factors affecting their population dynamics. RLKIs have a limited distributions and breed only on colonies with access to deep ocean basin waters [13]. Consequently, it has been postulated that RLKIs are highly specialized nocturnal foragers preying on vertically migrating deep water fish (i.e. myctophids) [14–18]. However, results of a recent study suggest that such a foraging specialization might be limited to the reproductive season only, as post-reproductive RLKIs distribute onto the shallow Bering Sea shelf and feed on non-myctophid prey during daylight hours [19]. A fine-scale behavioral study on diel patterns, location, and persistence of RLKI foraging behavior during the breeding season, when birds’ energy requirements are elevated [20], is important to better understand their population responses to changing conditions in the Bering Sea pelagic ecosystem.

A combination of bio-logging techniques such as GPS-tracking and accerelometry enabled us to characterize the fine-scale location and detailed foraging behavior of marine predators under natural conditions. GPS loggers have been utilized to specify foraging ranges [21,22], and the accelerometers have been used to classify at-sea behavioral patterns [23,24] at a fine scale (i.e. to distinguish between flying among prey patches and actively foraging). Specifically, acceleration-based information is suitable for characterizing/classifying multiple behavioral states and quantifying activities which is not possible from only GPS-based information. Recent miniaturization of these devices has enabled us to investigate the foraging ecology of medium to small sized seabirds [25].

The objective of this study was to investigate whether RLKIs show elevated foraging activity at night over the ocean basin during the breeding season, as anticipated from previous observational and dietary studies. We used bio-logging techniques and the collection of regurgitated diet samples to achieve this objective.

## Materials and Methods

### Ethics statement

This study was conducted under all required federal, state, and special use permits, and in accordance with the University of Alaska Fairbanks IACUC (assurance # 471022–2). All live-capture and tagging works were conducted following the Federal Fish and Wildlife Permit issued by the U. S. Fish and Wildlife Service (permit # MB70337A-3) and the Scientific Permit issued by the State of Alaska (permit # 13–079). Details of collection and sampling methods are provided in "Deployment of data loggers" in "Materials and Methods". Instrument weight was up to ~4% of average RLKI body mass (373 ± 29.3 g, *n* = 19) for GPS loggers, which exceeds the recommended 3% threshold for other seabird groups [26]. However, closely related and morphologically similar black-legged kittiwakes are able to carry tags >3% of their body mass without significant detrimental effects on their foraging behavior [27]. In this study, among 19 instrumented RLKIs, only one individual lost a chick during the instrument deployment period (3.8 ± 3.1 days). The proportion of chick loss (5.3%) for the instrumented birds was comparable to the average chick loss rate of non-instrumented birds (5.4% during 3.8 days; [28]).

### Study site

The field study was conducted in the southeastern Bering Sea on St. George Island, home to the largest colony of RLKI in the world (~220,000 birds [29]). We captured birds on their nests at two locations: High Bluffs (56°36’N, 169°40’W) and Village Cove (56°36’N, 169°32’W), both on the northern side of the island. The study period was from 22nd July to 5th August 2013, and instruments were deployed on birds guarding chicks. During the study period, sunrise and sunset ranged between 07:02–07:28 and 23:47–23:20 local time. The start and end of nautical twilight (when the sun is less than 12° below the horizon) ranged between 04:30–05:30 and 02:20–01:20. We defined the time between sunrise to sunset as “daytime”, and the time between sunset and the next sunrise as “nighttime” which includes dusk (sunset to end of nautical twilight), dark night (end of nautical twilight to start of nautical twilight) and dawn (start of nautical twilight to sunrise). The mean proportion of daytime and nighttime in a day was 68.0% (66.1% at minimum to 69.8% at maximum) and 32.0% (30.2% at minimum to 33.9% at maximum), respectively.

### Deployment of data loggers

We deployed either a GPS logger or an accelerometer on each study bird. We did not deploy both GPS loggers and accelerometers on the same individual to reduce the loading weight. Two types of GPS loggers (GiPSy 2 with chip antenna and 500mAh rechargeable battery: 47x24x11 mm, and GiPSy 4 with chip antenna and 500mAh rechargeable battery: 46x27x11 mm, TechnoSmart, Rome, Italy) were deployed on 19 individuals. The logger was housed in heat-shrink tubing for water-proofing. The loggers were attached to the medial portion of the back of the birds with strips of Tesa® tape, and cyanoacrylate glue (Loctite® 401) to secure the end of the tape. The total weight of the GPS loggers including the heat-shrink tubing and tape were 15.1g for GiPSy 2 and 16.3g for GiPSy 4, which correspond 4.1 ± 0.3% and 4.4 ± 0.4% of RLKI body mass. The loggers were set to record a position every second (GiPSy 2) and every 6 seconds (GiPSy 4). The GPS loggers had neither depth nor temperature sensors.

Accelerometers (ORI-380 D3GT: housed in a cylindrical container, 12 mm diameter, 45 mm length, mass 10 g, which corresponds to 2.7 ± 0.2% of RLKI body mass, Little Leonardo, Tokyo, Japan) were deployed on 5 individuals to record their body movements. The loggers were attached to the lower portion of the chest (near the lower end of sternal keel). The attachment method was the same as for the GPS loggers. Tri-axial acceleration (heave, surge and sway) was recorded at a rate of 20 Hz (every 0.05 s). Depth (at a resolution of 0.1 m) and temperature (at a resolution of 0.1°C) data were recorded every second.

The birds were captured with a 5 m noose pole, and body mass was measured to the nearest 5 g by a Pesola® balance. Handling time was less than 11 min. The birds were recaptured between 2 to 6 days (in one case 13 days) after the tag deployment. The loggers were removed and the data were downloaded to a laptop computer.

### GPS tracking

GPS locations were first re-sampled every 1 min from all recorded fixed locations. If the ground speed exceeded 20 m/s (cf. ~80 km/h [27]) the location was considered erroneous and was removed. This filtering process picked up 26 erroneous points out of 10,153 total points, both at nest sites and during foraging trips. We did not smooth or interpolate to fill the missing points to reduce uncertainty in the flight speed. Next we established a cut-off value of ground speed (3 m/s, see results) to discriminate flight and non-flight behaviors [30]. A RLKI is likely to reduce movement speed during foraging, due to contact with the prey swimming near the ocean’s surface [10]. Therefore, non-flight behaviors obtained with GPS include potential foraging activity and resting at the surface.

We mapped the locations where the flight and non-flight behaviors occurred over the bathymetric chart (e-topo 1 topographic map) by Arc View® v. 8.2. The marine habitats were defined based on bathymetry as follows: on-shelf: 0–200 m bottom depth, shelf break: 200–1,000 m bottom depth, ocean basin: >1,000 m bottom depth [27], and on-land: >0 m altitude. We measured proportions of foraging trips spent in the on-shelf, shelf break and ocean basin areas. Then we tested whether non-flight behaviors had occurred in a particular area more often than expected if those were distributed evenly over the trip with respect to bathymetry. We also investigated the diel pattern of flight and non-flight behaviors.

### Accelerometry

The behavior of RLKI during trips was analyzed using the heave (dorsal-ventral axis of the body) acceleration records with the Ethographer package [31] of Igor Pro v. 6.0 software (Wave Metrics Inc., Lake Oswego, OR, USA). We categorized three types of behaviors (flight, resting on water, foraging) following previous studies on closely-related black-legged kittiwakes (*Rissa tridactyla*) [11,27,32,33] or at sea behavioral observations of RLKIs [10]. The detailed method of the categorization is described in the S1 Text. We examined the diel patterns and persistence of these three behavioral categories.

### Foraging trip parameters

The trip duration was calculated using both the GPS and acceleration data. The maximum distance from the colony during a foraging trip was calculated based on GPS data. The definitions of foraging trip, trip duration and trip distance are provided in the S1 Text. We categorized an overnight trip as started on one day and ended the next (i.e. crossed the local midnight time) [27]. We categorized all other types of trips as day trips. Also, we calculated the proportion of time spent on foraging trips (during day and night) in relation to the total duration recorded.

### Dietary analyses

We opportunistically collected food regurgitated by birds at deployment and/or retrieval of data loggers. Diet samples were stored frozen until lab analyses, when they were weighed to the nearest 1 g, visually sorted and prey were identified to the lowest possible taxonomic level. We collected otoliths when fish skulls were found. The shape and size of the otoliths were inspected under a microscope to identify fish species by referring to a fish otolith catalog [34]. The standard length of the identified fish was estimated from the otolith size using available equations on the relationship between body- and otolith-length [34].

### Statistics

The data were analyzed with generalized linear mixed models (GLMM). We compared (1) durations between the three behavioral categories, and (2) relative proportions of the three behavioral categories between day and night. In the first model, behavioral type was set as a fixed factor, and trip and bird identity were included as random factors. In the second model, time of day (day or night) and trip identity were included as fixed factors, and bird identity was set as a random factor. The models with and without the effect of fixed factors were compared using Likelihood Ratio Test (LRT) [35]. A Gamma or binomial error distribution was used for analyzing duration of each behavior type and proportion of each behavior category, respectively. We used the “lme4” package in R® 2.7.0 software [36]. Data are presented as mean values ± SD, with significance set at the 0.05 level.

## Results

### Data recovery and foraging trip characteristics

We recaptured and recovered GPS loggers from 15 out of 19 birds. We could not recapture the other four birds because they became leery of the noose pole as their chicks grew older (three individuals) or, in one case, the logger was lost. Among the 15 GPS loggers, 10 tags failed to record locations properly, most of which occurred early in the study period. The main reason of the positioning failure could be malfunction of the antenna and/or influence of local topography. As a result, data for 14 trips from 5 individuals (all from the High Bluffs study site, 5 trips per bird at maximum) were available. Trip duration of 9 trips and trip distance of 5 trips out of the 14 were 14.4 ± 5.8 h and 107.0 ± 55.4 km, respectively. We were not able to measure the duration of the other 5 trips and the distance of the other 9 trips due to large gaps in the GPS positioning. Among the 9 trips, 2 were day trips (trip duration: 12.3 and 5.4 h; trip distance: not available) and 7 were overnight trips (trip duration: 15.9 ± 5.2 h; trip distance of 5 trips: 107.0 ± 55.4 km). During the night, birds spent most of their time performing foraging trips (86.5%), whereas during the day they spent somewhat less time allocated to foraging trips (67.3%).

We were able to recapture 4 out of 5 birds with accelerometers still attached. The data for 7 trips (2 trips per bird at maximum) were available, and 5 trips were fully covered (from a bird’s departure to arrival from/to the colony). Mean trip duration of the 5 trips was 19.0 ± 5.6 h. All of the 7 trips were overnight trips. Again at night birds spent a majority of their time allocated to foraging trips (84.1%), compared to during the day (58.1%).

### GPS tracking

**Fig 1:**
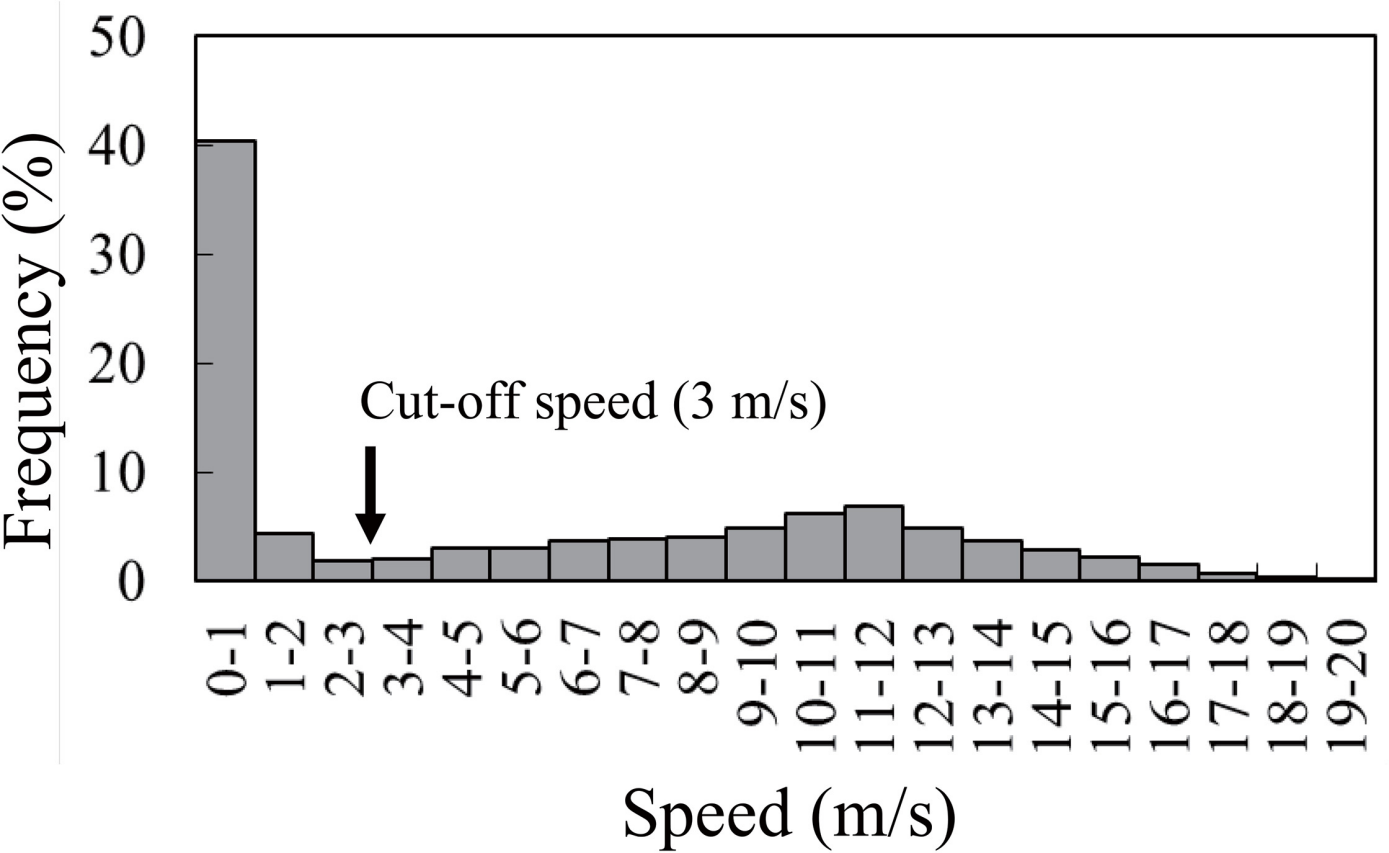
Frequency distribution of ground speed (m/s) of red-legged kittiwakes as obtained by GPS loggers. The black arrow (3 m/s) shows a cut-off value discriminating flight and non-flight behaviors.

**Fig 2:**
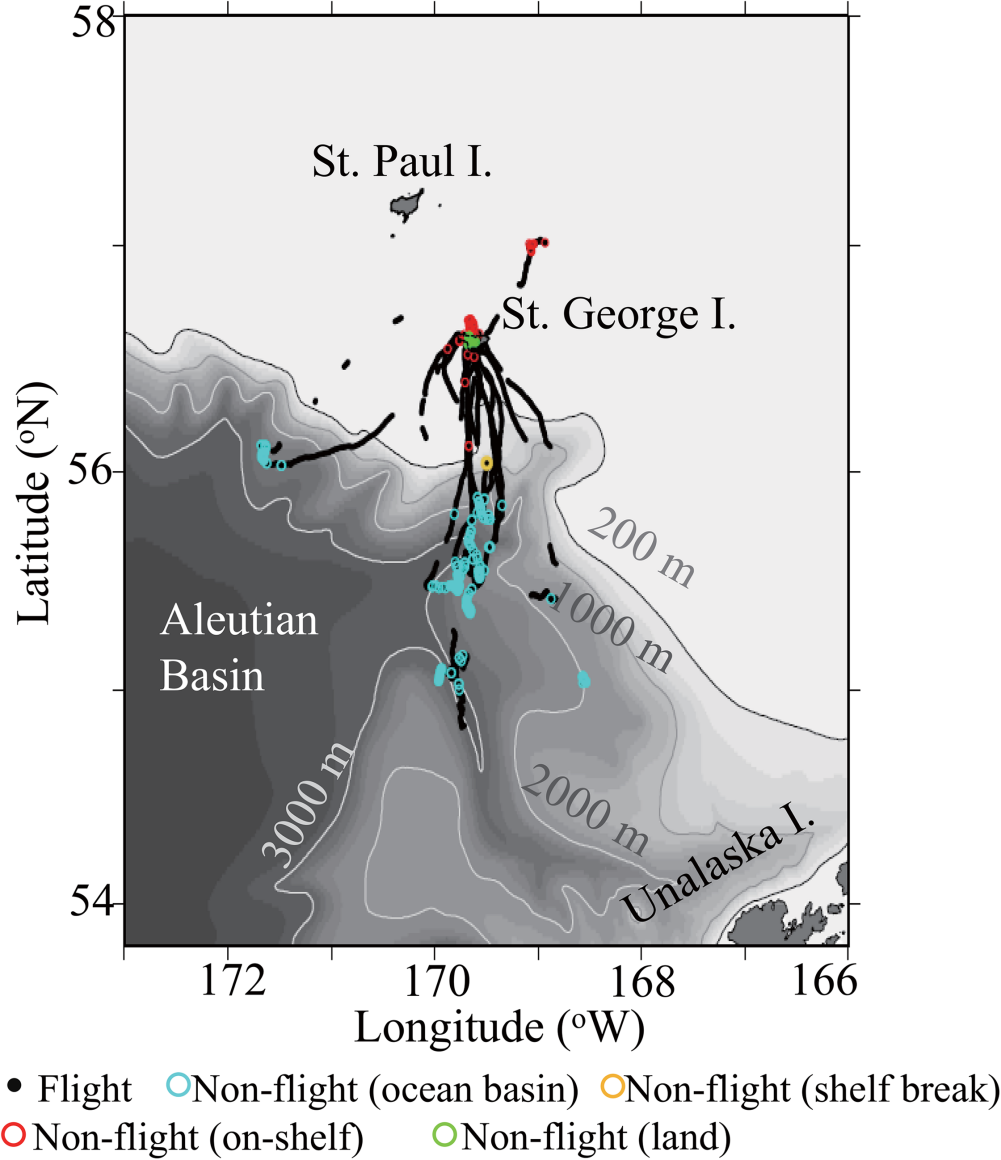
Locations of different types of behavior of red-legged kittiwakes as revealed by GPS loggers in relation to bathymetry. The blue dots represent flight behavior, the open light blue circles represent non-flight behavior (including foraging and resting on water) that occurred in ocean basin (where bottom depth >1,000 m), the open yellow circles represent non-flight behavior that occurred on the shelf break (bottom depth: 200 to 1000 m), and the open red circles represent non-flight behavior that occurred on the shelf (bottom depth: 0 to 200 m). The open green circles represent bathing behavior which occurred on land.

The GPS loggers fixed 5,144 points (excluding 20 erroneous points detected by the speed filter). Ground speed was calculated with 5,053 continuously fixed points among them (Fig 1, S1 Dataset). We regarded the points with speed less than the cut-off value 3.0 m/s (in total 2,347 points) as potential foraging and resting locations. Most of the foraging/resting behavior (82.5%) occurred in the ocean basin, which was more frequent than expected (65.2%) if it was distributed evenly over the trip (Table 1: $\chi_{3}^{2}$= 285, *P* < 0.001, Fig 2). Specifically, the Pribilof Canyon was one of the hot-spots where the foraging/resting locations were concentrated (Fig 2).

**Table 1:** Frequency of different types of behavior of red-legged kittiwakes in relation to bathymetry as revealed by GPS loggers.

Habitat type	Total locations	Foraging/resting locations
On land	391 (7.7%)	100 (4.3%)
On-shelf	1078 (21.3%)	308 (13.1%)
Shelf break	290 (5.7%)	2 (0.1%)
Ocean basin	3294 (65.2%)	1937 (82.5%)

### Accelerometry

**Fig 3:**
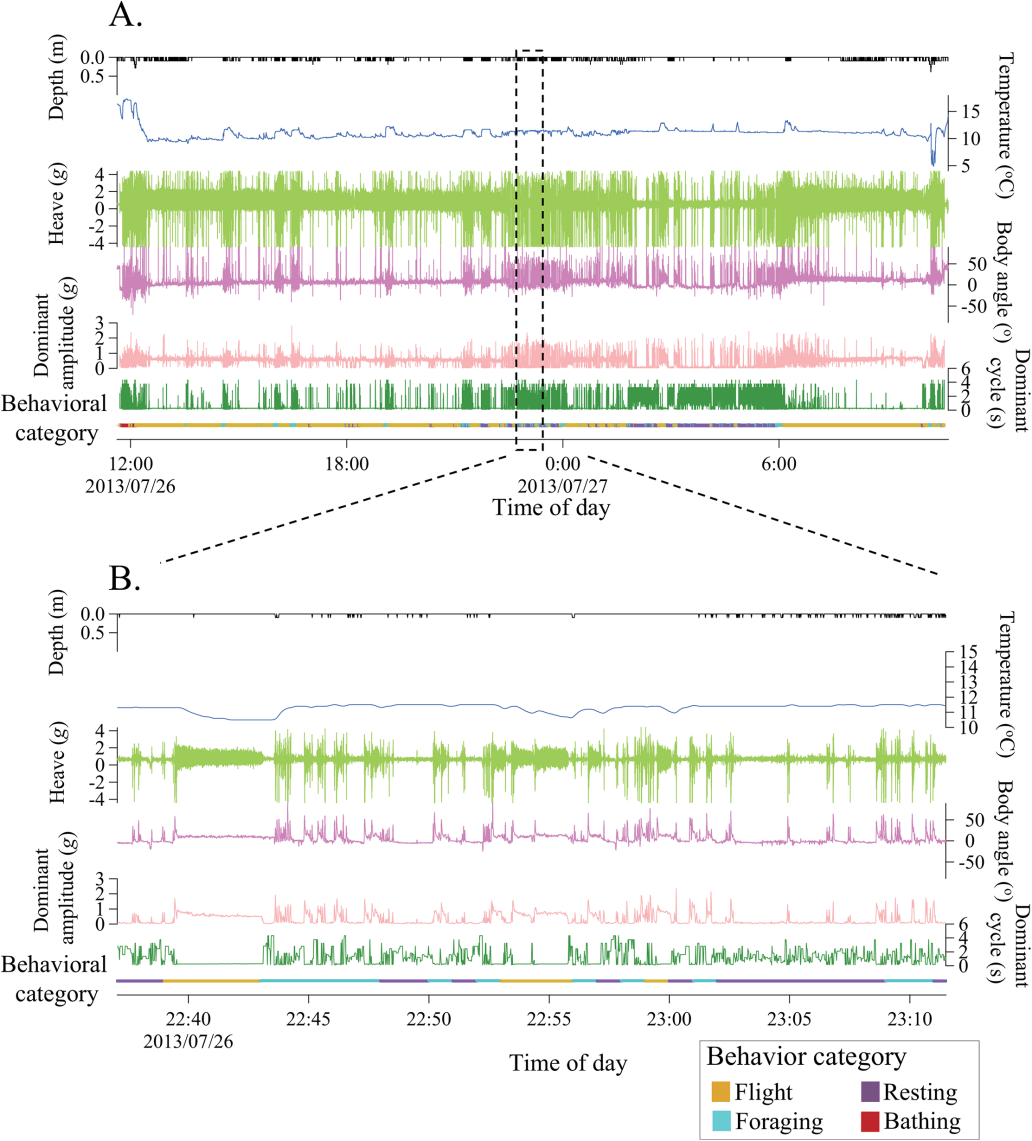
Behavioral patterns of red-legged kittiwakes as revealed by accelerometers. Panel A shows a time series of depth, heave acceleration, body angle, dominant amplitude of the heave acceleration, dominant cycle of the heave acceleration, and behavioral categories determined by accelerometry (the colors correspond to each behavioral category). Panel B shows enlarged records from panel A.

Diel behavioral patterns of RLKIs during a trip was determined by *k*-means cluster analysis (Table 2, Fig 3, S2 Dataset) and showed that RLKIs were likely to forage at the surface interposed by periods of resting on the water and flying (Fig 3B). The mean duration differed between the three types of behavior (2.4 ± 2.9 min, *n* = 298; 3.6 ± 4.2 min, *n* = 187; and 24.2 ± 53.1 min, *n* = 267 for foraging, resting on water and flight, respectively, GLMM with LRT, χ^2^ = 735, *P* < 0.001). Dives (>50 cm depth) were not recorded for any birds during the study period.

**Table 2:** Behavioral categories of red-legged kittiwakes as determined by accelerometry.

		Average duration of each behavioral element during one minute (*n* = 7 trips)		
Behavioral category	*n* (min)	Regular wing flapping	Irregular wing flapping	Non-active behavior
Flight	6468	57.8 ± 4.6 s	1.4 ± 3.4 s	0.7 ± 2.5 s
Resting on water	670	2.8 ± 6.1 s	10.6 ± 7.9 s	46.7 ± 9.1 s
Foraging	763	13.1 ± 11.6 s	32.5 ± 12.5 s	14.4 ± 9.5 s

### Activity time budget

**Fig 4:**
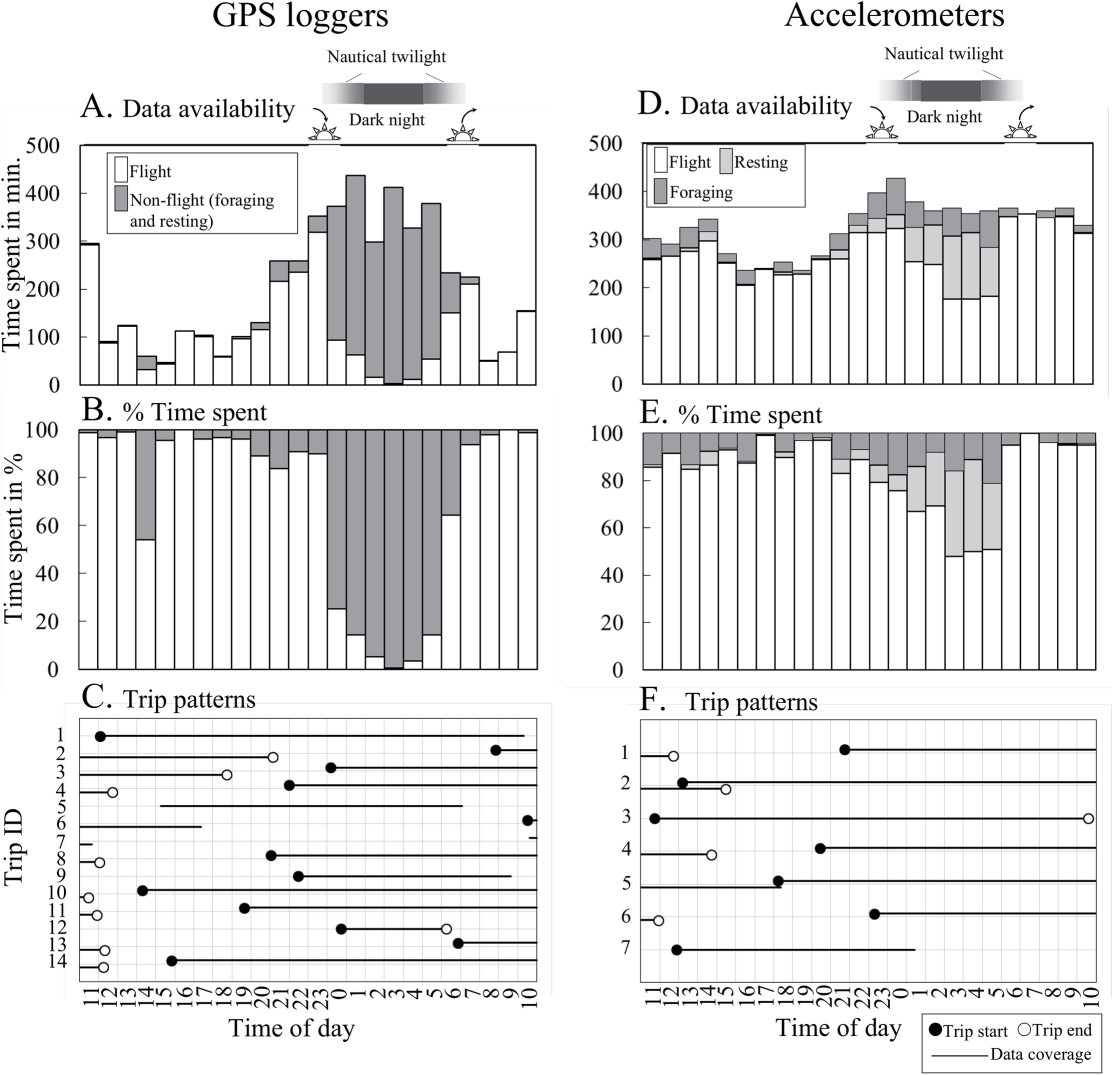
Diel behavioral patterns of red-legged kittiwakes. The left panels (A, B, and C) represent the data from GPS loggers, and the right panels (D, E, F) represent the data from accelerometry. Sunset, sunrise, twilight and dark night times are shown at the top. The top panels (A and D) show data available for each hour of a day, the middle panels (B and E) show % data occurrence, and the bottom panels (C and F) show patterns of complete foraging trips. In the bottom panels, solid lines represent the range when the data were obtained for each trip. The trip ID is shown by the vertical axis. The closed and open circles represent the timing of the start and the end of the trips, respectively.

Activity time budget with respect to the time of day are shown in Table 3 and Fig 4. There was variation in the number of fixed points in regard to the time of day for the GPS birds (Fig 4A). Proportion of non-flight behaviors was higher during the night compared to those during the day (Fig 4B, Table 3: GLMM with LRT, χ^2^ = 1159, *P* < 0.001).

**Table 3:** Activity time budget of red-legged kittiwakes during foraging trips as revealed by GPS loggers and accelerometers.

	GPS tracking (*n* = 14 trips)		Accelerometry (*n* = 7 trips)		
Behavior category	Flight	Non-flight	Flight	Resting on water	Foraging
No. of data points (min)	2706	2247	6468	667	714
Proportion of total (%)	65.6 ± 28.8	34.4 ± 28.8	82.0 ± 8.2	8.6 ± 6.5	9.3 ± 3.8
Proportion during daytime (%)	95.5 ± 5.7	4.5 ± 5.7	91.7 ± 5.0	1.7 ± 2.7	6.5 ± 3.0
Proportion during nighttime (%)	65.6 ± 32.3	34.4 ± 32.3	65.0 ± 16.5	20.8 ± 14.3	14.1 ± 7.1
GLMM (B) & LRT^a^	χ^2^ = 687, *P* < 0.001	χ^2^ = 1159, *P* < 0.001	χ^2^ = 74, *P* < 0.001	χ^2^ = 575, *P* < 0.001	χ^2^ = 58, *P* < 0.001

^a^ Generalized linear mixed model (a binomial distribution) with likelihood ratio test was used. Trip identity and bird identity were included as fixed factor and random effect, respectively. See "Statistics" in the "Materials and Methods".

There was less diurnal variation in data availability for the accelerometry birds (Fig 4A and 4D). The proportion of both foraging and resting on the water behaviors were higher during the night compared to the day (Fig 4E and Table 3: GLMM with LRT, χ^2^ = 58, *P* < 0.001 for foraging, GLMM with LRT, χ^2^ = 575, *P* < 0.001 for resting on the water).

### Dietary analyses

We obtained 22 regurgitated diet samples (average mass 14.7 ± 10.8 g) from 19 birds (including 3 individuals with diet samples obtained from both before and after a foraging trip). All diet samples included myctophids (i.e. “lampfish”, 98.4 ± 2.4% by wet weight), and 55 individual otoliths were collected from 18 diet samples (1 to 9 otoliths per sample). Among them, 35 otoliths (one per fish skull) were inspected under a microscope to identify the species [34]. Six northern lampfish (*Stenobrachius leucopsarus*) and one pinpoint lampfish (*Nannobrachium regale*) were identified. The remaining 28 fish were identified as *Stenobrachius* sp. (either *S*. *leucopsarus* or *S*. *nannochir*). The standard lengths of the fish estimated by otolith length [34] were 7.5 ± 0.5 cm for *S*. *leucopsarus* (*n* = 6) and 12.6 cm for *N*. *regale* (n = 1). Other prey items included amphipods (27.3% occurrence and 0.8 ± 1.7%, wet weight), cephalopods (13.6% occurrence and 0.8 ± 2.1%, wet weight), decapods (shrimps: 9.1% occurrence and <0.1%, wet weight) and euphausiids (4.5% occurrence and <0.1%, wet weight).

## Discussion

This study investigated the foraging characteristics of red-legged kittiwakes breeding in the southeastern Bering Sea, using accelerometry (*n* = 4 birds), GPS tracking (*n* = 5 birds), and dietary analyses (*n* = 19 birds). Admittedly sample sizes were small, however foraging patterns of chick-rearing kittiwakes were still apparent: (i) foraging activity increased at night (including dusk, dark night and dawn), (ii) most non-flight (foraging or resting) behavior (>80%) occurred in ocean basin regions, (iii) no dives deeper than 50 cm were recorded during trips. These behavioral patterns were consistent among focal individuals and illustrate the nocturnal surface foraging of RLKIs in the ocean basin region, as previously hypothesized by ship-based observations of RLKIs [14]. These behavioral patterns may be related to the distribution and availability of RLKI’s primary myctophid prey in several ways.

First, the dominant myctophid species found in diet samples (*S*. *leucopsaurus* and *N*. *regale*) are commonly distributed only in deep ocean regions such as the Aleutian Basin [37,38]. These species have been documented in the upper 50 m depth at night [38]. RLKI may concentrate their foraging efforts in the ocean basin at night (Table 1, Table 3, Figs 2 and 4B and 4E), responding to horizontal (basin-scale) and vertical (diel-scale) distribution patterns of these myctophid prey. Trip duration and maximum trip distance of RLKIs (14.4 ± 5.8 h and 107.0 ± 55.4 km) were longer and/or further than day trips of sympatric black-legged kittiwakes (5.2 ± 0.8 h and 33.5 ± 6.5 km, over the on-shelf region [27]), but similar to their overnight trips (18.9 ± 2.3 h and 134.5 ± 13 km, over ocean basin regions [27]). A previous study on prey species of kittiwakes demonstrated that pelagic myctophids have higher energy content compared to other types of prey [27]. Trips to the ocean basin may require more energy than trips over the on-shelf region, but RLKIs may be rewarded by obtaining energy-rich prey.

Second, birds foraged for a relatively short duration (2.4 ± 2.9 min) compared to the duration of flights between foraging patches (24.2 ± 53.1 min) or resting on the water (3.6 ± 4.2 min). Previous studies of other surface feeding seabirds reported longer duration of foraging events ranging from 3 to 30 min for albatrosses [39] and from 23 to 88 min for fulmars [40]. The ephemeral nature of the RLKIs foraging behavior suggests that myctophids ascend to the surface for a limited time at a short (~min) scale, even though they may form a dense subsurface layer [41]. Limited prey availability may force RLKIs to fly and land frequently in the foraging habitat (as shown in Fig 3) to detect floating myctophids, and may possibly rely on the myctophid’s bio-luminescence as a visual cue [42]. There is increasing evidence that myctophids are a key prey species for pelagic top predators [17], but the processes that affect the availability of these prey on the surface are still not known. Temporal variations in the local oceanographic conditions can affect persistence of prey patches near the surface and might be important to surface-feeding seabirds [43]. A recent study hypothesized that the existence of meso-scale eddies, which typically develop in the Aleutian Basin [44], is a process that may affect the distribution of myctophids near the surface [45]. Furthermore, our results suggest that submarine canyons located south of the study colony with a prominent upwelling of deep ocean water masses to the surface layers [46] may be one of the main foraging habitats for RLKIs feeding on myctophids (Fig 2). The submarine canyon enhances water mass exchange between the Aleutian Basin and the Bering Sea continental shelf and forms a productive habitat for plankton and fishes [46]. It is possible that myctophids come up to the surface to feed on enriched plankton, or they might be passively advected to the surface by tidal currents and/or upwelling.

A recent study of RLKIs during the non-breeding season [19] showed different foraging patterns than those observed in our study. During the non-breeding seasons, RLKIs mainly foraged in the Bering Sea shelf regions where myctophids are rarely found [19,37,38]. In addition, non-breeding RLKIs foraged mostly during the day [19]. Such contrasting foraging patterns suggest RLKIs forage on different types of prey and in different habitats between the breeding and non-breeding seasons.

Myctophid distribution patterns may also explain inter-seasonal differences in RLKI foraging behavior. Myctophids show seasonal changes in their vertical migration pattern [47], and their distribution fluctuates annually [48]. The reasons for seasonal/inter-annual variability of myctophid distribution are not well understood but presumably reflect changes in the physical environment and/or the availability of zooplankton [47,48]. It would be important to identify the local marine features and seasonal/annual variability that affect vertical and horizontal distributions of myctophids, which may be a key factor to understand the foraging success of chick-rearing RLKIs.

One of the important factors promoting the nocturnal foraging of RLKIs may be the inter-specific competition with black-legged kittiwakes, an abundant surface feeder (209,000 RLKI and 619,000 black-legged kittiwakes breed in the eastern Bering Sea [49]). On St. George I., population trends show that RLKIs declined rapidly from 1976 regime shift until the late 1980s and then recovered from 1989 regime shift, whereas black-legged kittiwakes did not show such significant changes [28,29]. The black-legged kittiwakes perform on-shelf foraging trips more frequently and feed on more various prey [45] in contrast with the RLKIs. It is possible that differences in foraging characteristics between a specialist RLKI and a more generalist black-legged kittiwake [45] played a role. Further investigation into inter-specific comparison of foraging behaviors between RLKIs and black-legged kittiwakes during the breeding season (but see [19] for the inter-specific comparison during non-breeding season) may allow us to better understand the underlying processes of co-existence of these congeneric top predators and their different responses to environmental change [29,50].

In conclusion, we used a combination of GPS tracking, accerelometry, and diet studies to show that red-legged kittiwakes breeding in the southeastern Bering Sea, foraged on deep water myctophids at the surface, mostly in the ocean basin regions, and mainly at night. We suggest that clear diel patterns and the ephemeral nature of red-legged kittiwake foraging activity reflect the availability of their myctophid prey, and future studies focused on the ecology of these not well-known deep ocean fishes might be important to our better understanding of the bottom-up effects on red-legged kittiwake population.

## Supporting Information

S1 Dataset Location and behavioral type data as obtained by GPS loggers. (XLS)

S2 Dataset Behavioral type data as obtained by accelerometers. (XLS)

S1 Text Supplementary Methods. (DOC)
