# Supplementary material for: Nocturnal Foraging by Red-Legged Kittiwakes, a Surface Feeding Seabird That Relies on Deep Water Prey During Reproduction
Source: PLoS One. 2015 Oct 14;10(10):e0138850. doi: 10.1371/journal.pone.0138850 (PMC4605634; doi:10.1371/journal.pone.0138850)
Supplement: S1 Text — (DOC) [file pone.0138850.s003.doc]

**SUPPORTING INFORMATION**

**S1 Text. Supplementary Methods**

Nobuo Kokubun, Takashi Yamamoto, Dale M. Kikuchi, Alexander Kitaysky, Akinori Takahashi

**Accelerometry**

We classified three behavioral elements based on the magnitude and persistence of dominant amplitudes and cycles of heave acceleration. The three behavioral elements include: (i) regular wing flapping, with a dominant amplitude ≧0.1 *g* (where “*g*“ denotes the gravity acceleration) and continues 5 s or longer with dominant cycles of heave acceleration at 0.1-0.5 s, which presumably reflects the average wing beat cycle of RLKIs (0.25 s [1]), (ii) irregular wing flapping, with a dominant amplitude ≧0.1 *g* but interrupted within 5 s, and (iii) non-active, with a dominant amplitude <0.1 *g*. We counted these three behavioral elements, with a precision of one second during each min of observations, to categorize three behaviors (flight, resting on water, foraging) following the previous studies on closely-related black-legged kittiwakes (*Rissa tridactyla*) [2,3,4,5] or at sea behavioral observations of RLKIs [1]. The three behaviors were classified using *k*-means clustering of the proportion of three behavioral elements in each minute as follows: (1) flight, characterized by dominance of regular wing flapping, (2) resting on water, characterized by dominance of non-active behavior, and (3) foraging, characterized by dominance of irregular wing flapping (see Table 1). The categorization of three behaviors every min enabled us to match the behavioral time scale determined by accelerometry with those determined by GPS tracking. The *k*-means clustering analysis was made by ‘cluster’ library in R 2.15.1 software [6].

Body angle was calculated using a low-pass filter of 1 Hz on surge acceleration [7]. The standard body angle (0^o^) was defined when the birds were likely resting on the surface. The body angle was used for determining nest attendance.

**Trip parameters**

For birds with GPS loggers, we defined “trips” as any time period when the bird was away from the colony for more than 1 hour. We defined the departure time of trips as the time when the bird went beyond 100 m from the nest site and the arrival time when the bird returned within 100 m of the nest site. We defined the trip distance as the maximum distance from the colony during a trip. If the missing points exceeded 50% of the trip, the data were not used for calculation of trip distance. Trip data used for the calculation of trip distance covered 73.5 ± 13.2% (ranging from 59.9 to 93.0%, *n* = 5 trips) of the total recorded period. These data covered 82.8 ± 14.0% (ranging from 62.7 to 98.8%) of the middle half part (from 25 to 75%) of the recorded period.

If non-flight behaviors occurred in the interior parts of the island (detectable by comparing the location with topography), we assumed a bird was bathing in freshwater, which is common among kittiwakes [1]. We excluded the bathing from analyses of diel behavioral pattern as it is not related to foraging behavior.

For birds with accelerometers, we determined the departure and the arrival timing of trips based on an assumption that temperature and body angle may change between the two states, on-nests and during foraging trips: i.e. birds standing on the nest showed higher temperature (15.7 ± 3.1^o^C for on-nest site, *n* = 9 trips; 11.7 ± 0.7^o^C for during trips, *n* = 7 trips) and higher body angle (from surge acceleration: 27.2 ± 11.3 ^o^ for on-nest site, *n* = 9 trips; -4.6 ± 6.0 ^o^ for during trips, *n* = 7 trips) possibly due to heat from the chicks and standing posture. Again, foraging trips were counted only when the bird was away from the nest for more than one hour. In the cases when resting on the water or foraging behaviors were recorded right after/before the trip departure/arrival time and where the environmental temperature was >15^o^C, we regarded them as “bathing in fresh water” and excluded these points from the analyses of diel behavioral patterns. The threshold 15^o^C was determined by the August sea-surface temperature (SST) obtained from satellite images (10.1 ± 1.0^o^C range 6.7^o^C to 14.0^o^C). We used August SST (reanalyzed ‘SST, Blended, Global, EXPERIMENTAL Monthly Composite’ with 0.1^o^x0.1^o^ resolution, available at National Oceanic and Atmospheric Administration webpage http://coastwatch.pfeg.noaa.gov/erddap/griddap/erdBAsstamday.graph?sst) for our analysis. SST was spatially averaged in the area 54.9^o^N to 57.0^o^N, 168.6^o^W to 171.6^o^W (*n* = 640 data points), which covers the maximum foraging range of RLKI.

**References**

1. Byrd GV, Williams JC. Red-legged kittiwake (*Rissa brevirostris*). In: Poole A, editor. The birds of north America online. Ithaca: Cornell Lab of Ornithology; 1993.

2. Coulson JC. The Kittiwake. London: T & AD Poyser; 2011.

3. Paredes R, Harding AMA, Irons D, Roby DD, Suryan RM, Orben, RA, et al. Proximity to multiple foraging habitats enhances seabirds’ resilience to local food shortages. Mar Ecol Prog Ser. 2012 Dec; 471:253-269.

4. Daunt F, Benvenuti S, Harris MP, Dall’Antonia L, Elston DA, Wanless S. Foraging strategies of the black-legged kittiwake *Rissa tridactyla* at a North Sea colony: evidence for a maximum foraging range. Mar Ecol Prog Ser 2002 Dec; 245:239-247.

5. Chivers LS, Lundy MG, Colhoun K, Newton SF, Houghton JDR, Reid N. Foraging trip time-activity budgets and reproductive success in the black-legged kittiwake. Mar. Ecol. Prog. Ser. 2012 Jun; 456:269-277.

6. R Development Core Team. R: A language and environment for statistical computing. Vienna: R Foundation for Statistical Computing; 2012.

7. Sato K, Charrassin JB, Bost CA, Naito Y. Why do macaroni penguins choose shallow body angles that result in longer descent and ascent durations? J Exp Biol. 2004 Nov; 207:4057-4065.
